# Prognostic Value of Baseline Medications Plus Neutrophil-to-Lymphocyte Ratio in the Effectiveness of Nivolumab and Pembrolizumab in Patients With Advanced Non-Small-Cell Lung Cancer: A Retrospective Study

**DOI:** 10.3389/fonc.2021.770268

**Published:** 2021-11-08

**Authors:** Toshiki Ogiwara, Hitoshi Kawazoe, Saeka Egami, Hironobu Hashimoto, Yoshimasa Saito, Naomi Sakiyama, Yuichiro Ohe, Masakazu Yamaguchi, Tetsuya Furukawa, Azusa Hara, Yui Hiraga, Aya Jibiki, Yuta Yokoyama, Sayo Suzuki, Tomonori Nakamura

**Affiliations:** ^1^ Division of Pharmaceutical Care Sciences, Center for Social Pharmacy and Pharmaceutical Care Sciences, Keio University Faculty of Pharmacy, Tokyo, Japan; ^2^ Division of Pharmaceutical Care Sciences, Keio University Graduate School of Pharmaceutical Sciences, Tokyo, Japan; ^3^ Department of Pharmacy, National Cancer Center Hospital, Tokyo, Japan; ^4^ Department of Thoracic Oncology, National Cancer Center Hospital, Tokyo, Japan; ^5^ Department of Pharmacy, Cancer Institute Hospital, Japanese Foundation for Cancer Research, Tokyo, Japan; ^6^ Division of Drug Development and Regulatory Science, Keio University Faculty of Pharmacy, Tokyo, Japan

**Keywords:** nivolumab, pembrolizumab, baseline medications, neutrophil-to-lymphocyte ratio, prognostic score

## Abstract

**Background:**

Nivolumab and pembrolizumab are the standard treatments for patients with advanced non-small-cell lung cancer (NSCLC). While there are reports on several inflammatory indices and the prognosis of patients with cancer, no study has combined baseline medication with the neutrophil-to-lymphocyte ratio (NLR) to predict clinical outcomes. This study investigated the efficacy of baseline medications plus NLR to predict the effectiveness of nivolumab and pembrolizumab in a real-world clinical setting.

**Methods:**

We conducted a single-center retrospective observational study of consecutive patients with advanced NSCLC who received nivolumab or pembrolizumab as first-line, second-line, or beyond treatment between December 2015 and November 2018 at the National Cancer Center Hospital in Japan. Progression-free survival (PFS) and overall survival (OS) were estimated using the Kaplan–Meier method. The drug-based prognostic score for baseline medications plus NLR was weighed based on the regression β coefficients. The multivariable Cox proportional hazard model was used to assess the association between the prognostic score-stratified groups and survival outcomes.

**Results:**

In total, 259 patients were evaluated in this study. A prognostic score calculated from the baseline medications plus NLR was used to categorize the patients into good (score 0), intermediate (scores 1–2), and poor (scores 3–6) -prognosis groups. The multivariable Cox proportional hazard model revealed a significant association between the poor-prognosis group and reduced OS. The hazard ratio of OS was 1.75 (95% confidence interval: 1.07–2.99; *P* = 0.031). In contrast, no association between these prognosis groups and PFS was observed.

**Conclusions:**

The findings suggest that the baseline medications with nivolumab or pembrolizumab plus NLR could lead to progressively shorter survival outcomes in patients with advanced NSCLC and could be used as a prognostic index for poor outcomes. However, to ascertain the clinical application of these findings, these concomitant medications need further validation in a large-scale multicenter study.

## Introduction

The Global Burden of Disease 2016 study estimated the burden of tracheal, bronchial, and lung cancers at ~36.4 million (1). In elderly individuals, polypharmacy, commonly defined as taking five or more medications daily, leads to several negative health consequences (2). Recently, polypharmacy has been used as an indicator of poor-prognosis for older patients with advanced non-small-cell lung cancer (NSCLC) treated with immune checkpoint inhibitors (ICIs), including nivolumab, pembrolizumab, and atezolizumab (3). Therefore, clinicians must pay attention to drug-drug interactions among baseline medications and ICIs and provide patients with polypharmacy with safe and effective ICI treatment. The development of ICIs has led to a paradigm shift in cancer treatment, resulting in durable responses in patients with malignant tumors. However, no biomarker has been established to predict their effectiveness.

Recently, baseline medications that affect the effectiveness of ICIs have become a major controversial issue because of their positive and negative roles and inconsistency among reports. The response rate of ICIs is around 20%, and prospective clinical trials in which co-administration of bezafibrate or metformin may increase the response rate of ICIs are ongoing (4, 5). Fibrates, such as bezafibrate, a peroxisome proliferator-activated receptor agonist that promotes fatty acid oxidation, have been shown to improve T-cell anti-tumor activity and synergize with programmed cell death protein 1 (PD-1) blockade for tumor suppression (6). Metformin, a drug prescribed for type II diabetes, demonstrates an anti-tumor effect through several routes, including immune-mediated mechanisms (7). However, retrospective studies have reported that corticosteroid use prior to treatment initiation of ICIs diminishes the treatment outcome (8, 9). This mechanism is generally easy to understand because corticosteroids have immunosuppressive effects and a potential effect on T-cell function (10). Another retrospective study showed that the use of antibiotics prior to ICIs reduced treatment efficacy in patients with advanced NSCLC, renal cell carcinoma, and urothelial carcinoma (11, 12). The gut microbiome influences the effectiveness of ICIs (12). In addition, proton pump inhibitors (PPIs) and nonsteroidal anti-inflammatory drugs (NSAIDs) have been reported to affect the intestinal microbiota (13), and both antibiotics and PPIs have been reported to be associated with shorter survival and disease-free survival after treatment with ICIs (14). Statins have also been demonstrated to be associated with better response and longer time-to-treatment failure in patients with advanced NSCLC treated with nivolumab (15). Therefore, it is important to clarify the effects of these drugs. Several studies have evaluated the association between baseline medications and the clinical outcomes of ICI treatment (16–19). Additionally, several studies have reported the prognostic utility of baseline peripheral blood counts, such as the neutrophil-to-lymphocyte ratio (NLR) and platelet-to-lymphocyte ratio (20–22). Importantly, the impact of baseline medications and that of routinely available blood cell counts at baseline were evaluated separately in these studies. However, no study has examined the association between baseline medications and NLR and clinical outcomes of ICI treatment in the Japanese population. Taken together, we hypothesized that combining baseline medications and NLR could predict clinical outcomes of nivolumab and pembrolizumab in clinical practice. Therefore, this study aimed to clarify whether baseline medications plus NLR affect the effectiveness of nivolumab and pembrolizumab in patients with advanced NSCLC using real-world data.

## Materials and Methods

### Patients

This single-center, retrospective observational study was carried out at the National Cancer Center Hospital, a high-volume cancer center in Tokyo, Japan, using data retrieved from electronic medical records. The methodology adopted in this observational study adhered to the STROBE Statement (23) and was the same as that followed in previous studies conducted by our coauthors (24–26).

The inclusion criteria were as follows: 1) consecutive patients aged ≥ 20 years who were diagnosed with postoperative relapse or unresectable stage III and IV NSCLC; 2) patients who had received at least one course of nivolumab monotherapy (3 mg/kg bodyweight every 2 weeks until August 2018, and, thereafter, a dose of 240 mg/kg bodyweight every 2 weeks) or pembrolizumab monotherapy (200 mg/kg bodyweight every 3 weeks) administered as first-line, second-line, or beyond treatment between December 2015 and November 2018; and 3) patients without complications or prior history of chronic or recurrent autoimmune disease and interstitial pulmonary disease. The treatment schedule and follow-up were modified at the clinician’s discretion according to the toxicity profile of each patient. Clinic visits and imaging evaluations were conducted every 6–8 weeks, starting at treatment initiation, according to the Response Evaluation Criteria in Solid Tumors (version 1.1) criteria (27).

The exclusion criteria were as follows: 1) history of prior administration of any ICIs and/or investigational drugs as part of a clinical trial or at a previous hospital before the investigation period; 2) discontinuation of treatment due to death or hospital transfer during the first 6 weeks after ICI treatment initiation, or only the first cycle of ICI because of disease progression or adverse events; 3) lack of baseline laboratory data (within 1 week before ICI treatment initiation), and 4) study participation shorter than 6 weeks (i.e., patients who started treatment between October and November 2018).

The study protocol was approved by the National Cancer Center Institutional Review Board (approval number: 2019-305) in Japan. The study was conducted in accordance with the Declaration of Helsinki and the Ethical Guidelines for Medical and Health Research involving Human Subjects by the Ministry of Education, Culture, Sports, Science, and Technology and the Ministry of Health, Labour, and Welfare of Japan. Acquiring written or oral informed consent from participants was waived because of the retrospective nature of the study. Accordingly, we used an opt-out method through the official website of the National Cancer Center Hospital.

### Data Collection

Research members from Keio University retrieved the patients’ data from electronic medical records held at the National Cancer Center Hospital. Patients’ baseline age, sex, cancer stage, Eastern Cooperative Oncology Group performance status (ECOG PS), chemotherapy regimen, treatment line, and medication history were extracted from physician and pharmacist records within 30 days of oral or intravenous administration before the initiation of ICI treatment. In addition, the routinely available laboratory data before ICI treatment initiation and the date of progression and death at the time of ICI treatment initiation were also collected. Patient records were then anonymized prior to data analysis by three investigators. In the present study, baseline concomitant medications were categorized as follows: corticosteroids (≥ 10 mg or < 10 mg prednisone equivalent per day), PPIs (yes or no), antibiotics (yes or no), metformin (yes or no), fibrates (yes or no), statins (yes or no), and NSAIDs (yes or no). We focused on the NLR at baseline (defined as the most recent blood count within one week before ICI treatment initiation) as routinely available blood cell counts. NLR was calculated as the absolute neutrophil count divided by the absolute lymphocyte count. The end of the follow-up period was August 31, 2020.

### Endpoints

Progression-free survival (PFS) was defined as the period from the date of the first administration of ICIs to the date of documentation of disease progression or death from any cause. Overall survival (OS) was defined as the period from the date of the first ICI administration to the date of death from any cause. Patients without documented radiographic progression or who were still alive were censored on the date of the last follow-up.

### Statistical Analyses

The Kaplan–Meier method was used to estimate the PFS and OS. The log-rank test was used to compare the differences among the three groups. The drug-based prognostic score for baseline concomitant medication plus NLR at baseline was weighed based on the regression β coefficients according to a previous study by Buti et al. (16). Univariable and multivariable Cox proportional hazards models were used to assess the association between the three groups of good, intermediate, and poor-prognosis and survival outcomes. Potential explanatory variables concerning the patient background, including age (10-year intervals), ECOG PS (2 vs. 0–1), and treatment line (later-line vs. first-line), were included in the multivariable model as independent variables. These explanatory variables were determined based on the clinical judgment of our coauthors. Cox proportional hazard regression was also used to compute the predicted probabilities for death according to the computed score to estimate the Harrell’s concordance statistic. All statistical analyses were performed using JMP 15.0.0 and SAS 9.4 (SAS Institute, Inc., Cary, NC, USA). All *P*-values were two-sided, and the statistical significance was set at *P* < 0.05.

## Results

### Patient Characteristics

**Figure 1:**
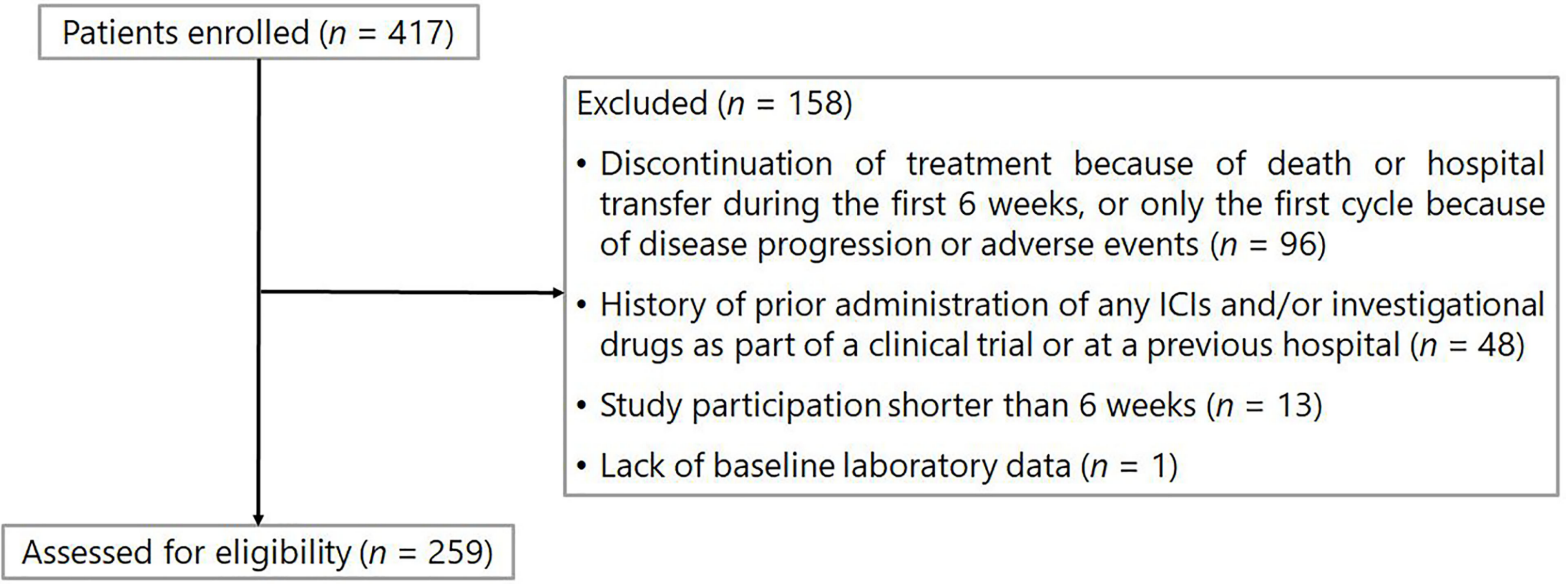
Patient enrollment flowchart. ICIs, immune checkpoint inhibitors.

The patient flowchart is shown in **Figure 1**. Of the 417 patients initially identified, 158 were excluded from the analysis based on the exclusion criteria. Data from 259 patients were included in the analysis. Baseline patient characteristics and baseline medications are listed in **Table 1**. The median age of the patients was 65 years [interquartile range (IQR): 56–70]. In total, 160 (61.8%) and 99 (38.2%) patients received nivolumab and pembrolizumab, respectively. A total of 231 (89.2%) patients were in good condition, with an ECOG PS of 0 or 1. The median values of the absolute neutrophil and lymphocyte counts at baseline were 4824 (IQR: 3531–6186) cells/mm^3^ and 1316 (IQR: 914–1697) cells/mm^3^, respectively. Thus, the median value of the NLR was 3.7 (IQR: 2.5–6.2).

**Table 1:** Baseline patient characteristics.

Characteristic	Total (*n* = 259)
Age (years), median (IQR)	65 (56–70)
Sex, *n* (%)	
Male	172 (66.4)
Female	87 (33.6)
ECOG PS, *n* (%)	
0	72 (27.8)
1	159 (61.4)
2	28 (10.8)
ICIs, *n* (%)	
Nivolumab	160 (61.8)
Pembrolizumab	99 (38.2)
Treatment line, *n* (%)	
First-line	64 (24.7)
Second-line	128 (49.4)
≥Third-line	67 (25.9)
Baseline concomitant medication, *n* (%)	
Corticosteroids	45 (17.4)
PPIs	108 (41.7)
Antibiotics	36 (13.9)
Metformin	6 (2.3)
Fibrates	2 (0.8)
Statins	18 (6.9)
NSAIDs	97 (37.5)
Peripheral blood counts at baseline (cells/mm^3^), median (IQR)	
Absolute neutrophil count	4824 (3531–6186)
Absolute lymphocyte count	1316 (914–1697)
NLR, median (IQR)	3.7 (2.5–6.2)

IQR, interquartile range; ECOG PS, Eastern Cooperative Oncology Group performance status; ICI, immune checkpoint inhibitor; PPI, proton pump inhibitor; NSAID, nonsteroidal anti-inflammatory drug; NLR, neutrophil-to-lymphocyte ratio.

### Endpoints

**Figure 2:**
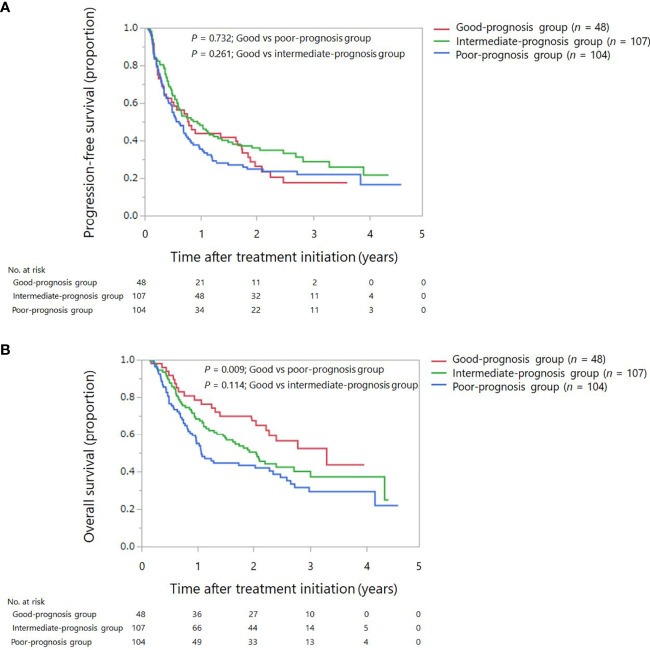
Kaplan–Meier survival curves for progression-free survival and overall survival among the three groups: good, intermediate, and poor-prognosis scores. **(A)** Progression-free survival. **(B)** Overall survival.

Overall, 188 events of disease progression and 142 deaths occurred. As shown in **Table 2**, we developed a drug-based prognostic score plus NLR based on regression β coefficients as follows: corticosteroids were assigned 2 points, whereas PPIs and antibiotics were assigned 1 point each. Additionally, we combined the original prognostic score of blood cell counts at baseline. NLR ≥ 3 was assigned 2 points, which was equivalent to that of corticosteroids. Thus, scores ranged from 0 to 6. Subsequently, we categorized patients into three prognostic groups according to previous studies as follows: score 0 (good-prognosis), scores 1–2 (intermediate-prognosis), and scores 3–6 (poor-prognosis). The Kaplan–Meier survival curves for PFS and OS among the three groups of good-, intermediate-, and poor-prognosis are shown in **Figure 2**. The poor-prognosis group was significantly associated with poorer OS than the good-prognosis group (*P* = 0.009), but not with PFS. As shown in **Tables 3**, **4**, the multivariable Cox proportional hazard model revealed that the poor-prognosis group was significantly associated with a reduced OS compared to the good-prognosis group. The hazard ratio of OS was 1.75 (95% CI: 1.07–2.99, *P* = 0.031). In contrast, no association between these prognosis groups and PFS was observed. The hazard ratios of PFS of the poor- and intermediate-prognosis groups were 0.97 (95% CI: 0.65–1.46, *P* = 0.879) and 0.81 (95% CI: 0.55–1.22, *P* = 0.308), respectively. Furthermore, the multivariable Cox proportional hazard model revealed that ECOG PS 2 and later-line treatment were significantly associated with a reduced OS. The hazard ratios of OS were 2.36 (95% CI: 1.40–3.80, *P* < 0.001) and 1.60 (95% CI: 1.06–2.51, *P* = 0.032), respectively. Similarly, the multivariable Cox proportional hazard model revealed that later-line treatment was significantly associated with a reduced PFS. The hazard ratio of PFS was 1.45 (95% CI: 1.02–2.09, *P* = 0.042). Applying the computed score to this population, the Harrell’s concordance statistic for OS was 0.634.

**Table 2:** Regression β coefficients for overall survival.

Variables	Regression β coefficients	Standard error
Corticosteroids	0.365	0.210
PPIs	0.250	0.169
Antibiotics	0.117	0.242
NLR ≥3	0.353	0.180

PPI, proton pump inhibitor; NLR, neutrophil-to-lymphocyte ratio.

**Table 3:** Univariable and multivariable Cox proportional hazard ratio of the prognostic score for progression-free survival.

Variable		No.	Event	Censored	Univariable analysis		Multivariable analysis	
					HR (95% CI)	*P*-Value	HR (95% CI)	*P*-Value
Group	Poor-prognosis	104	78	26	1.23 (0.92–1.64)	0.165	0.97 (0.65–1.46)	0.879
	Intermediate-prognosis	107	72	35	0.78 (0.58–1.04)	0.090	0.81 (0.55–1.22)	0.308
	Good-prognosis	48	38	10	1		1	
Age (10-year intervals)		–	–	–	0.90 (0.79–1.02)	0.086	0.92 (0.81–1.06)	0.242
ECOG PS	2	28	23	5	1.65 (1.03–2.50)	0.026	1.40 (0.85–2.19)	0.164
	0–1	231	165	66	1		1	
Treatment line	Later-line	195	149	46	1.47 (1.05–2.13)	0.032	1.45 (1.02–2.09)	0.042
	First-line	64	39	25	1		1	

ECOG PS, Eastern Cooperative Oncology Group performance status; HR, hazard ratio; CI, confidence interval.

**Table 4:** Univariable and multivariable Cox proportional hazard ratio of the prognostic score for overall survival.

Variable		No.	Event	Censored	Univariable analysis		Multivariable analysis	
					HR (95% CI)	*P*-Value	HR (95% CI)	*P*-Value
Group	Poor-prognosis	104	63	41	1.49 (1.07–2.08)	0.019	1.75 (1.07–2.99)	0.031
	Intermediate-prognosis	107	58	49	0.94 (0.67–1.31)	0.710	1.55 (0.95–2.61)	0.088
	Good-prognosis	48	21	27	1		1	
Age (10-year intervals)		–	–	–	0.91 (0.79–1.06)	0.227	0.98 (0.84–1.15)	0.777
ECOG PS	2	28	21	7	2.75 (1.68–4.29)	<0.001	2.36 (1.40–3.80)	<0.001
	0–1	231	121	110	1		1	
Treatment line	Later-line	195	116	79	1.66 (1.10–2.60)	0.020	1.60 (1.06–2.51)	0.032
	First-line	64	26	38	1		1	

ECOG PS, Eastern Cooperative Oncology Group performance status; HR, hazard ratio; CI, confidence interval.

## Discussion

The present study tested the hypothesis that baseline medications plus NLR could predict the clinical efficacy of nivolumab and pembrolizumab in clinical practice. Accordingly, the findings demonstrated that baseline medications plus NLR could reduce the effectiveness of nivolumab and pembrolizumab as first-line, second-line, or beyond treatment in patients with advanced NSCLC in a clinical setting. We showed that the poor-and intermediate-prognosis groups had reduced OS (increased risk: 75% and 55%, respectively) compared with the good-prognosis group. To our knowledge, this is the first study to report the association between baseline medications and routinely available blood cell counts at baseline and clinical outcomes of ICI treatment in the Japanese population using real-world data.

Previous studies have reported that a couple of baseline medications reduce the effectiveness of ICIs (8, 9, 11–19). For example, Buti et al. (16) reported that concomitant baseline medications using corticosteroids, antibiotics, and PPIs, at immunotherapy initiation, in patients with advanced cancer lead to progressively worse outcomes after ICI therapy. The study was the first to show that a drug-based prognostic score of baseline medications was significantly associated with survival outcomes in training (*n* = 217) and validation (*n* = 1012) cohorts. In another study, Buti et al. (17) reported that in patients with advanced NSCLC who received pembrolizumab (*n* = 950) as a first-line treatment, a drug-based prognostic score of baseline medication showed a predictive ability for survival outcome. Concordant with these studies, the present study demonstrated that NLR complimented the predictive ability of a drug-based prognostic score. In this population, baseline medications plus NLR had a higher predictive value than did the Buti’s scoring system and NLR (**Tables S1, S2**). In the present study, Harrell’s concordance statistic was similar to that reported by Buti et al. (16).

Interestingly, despite the difference in the treatment lines between the later-line and first-line treatments and the patients’ ethnicity among Japanese and Western populations, the prognostic utility of baseline NLR has been reported to be consistent (20–22). The present study combined baseline medications with the routinely available NLR at baseline as a novel prognostic score, and an NLR ≥ 3 was assigned 2 points as in previous studies (20–22). Rebuzzi et al. (20) reported that in patients with metastatic renal cell carcinoma who received nivolumab (*n* = 571) as a second-and further-line setting, the Meet-URO prognostic score of baseline NLR and clinical factors showed a predictive ability for survival outcome. However, when we categorized patients into three prognosis groups as follows: score 0 (good-prognosis), score 1–3 (intermediate-prognosis), and score 4–6 (poor-prognosis), the poor and intermediate-prognosis groups demonstrated reduced OS (increased risk: 72% and 62%, respectively) compared with the good-prognosis group. These findings suggest that baseline medications and NLR are key prognostic parameters. Once validated, these parameters could be used in the clinical setting, as they are easily available and do not require additional costs or setup. Hence, NLR was selected for the development of the prognostic score, based on Rebuzzi et al. (20).

The mechanism underlying the drug-drug interaction between baseline medications and ICIs has not been fully clarified, except in the case of corticosteroids. Studies have suggested that the diversity of the gut microbiota might influence the effectiveness of ICIs due to their association with immune status (12). Therefore, certain baseline medications that affect the gut microbiota, such as PPIs, NSAIDs, and metformin, might negatively impact the ICI treatment outcomes. For example, it has been shown that PPIs are frequently prescribed, with 25–70% of PPIs being prescribed inappropriately (28). Overuse of PPIs in geriatric patients requiring short-term treatment could lead to polypharmacy and its associated complications (28). The findings of the present and previous studies suggest that pharmacists should intervene and encourage clinicians to switch PPIs to alternative gastric acid suppressants or mucosa-protective agents on an as-needed basis.

On the contrary, the mechanism underlying the correlation of higher NLR with the survival outcome associated with ICI treatment has not yet been fully clarified. Interestingly, NLR ≥ 3 or 4 has been frequently reported in previous studies (20–22). However, it has been shown that ICIs interrupt immune suppression and activate CD8-positive T lymphocytes in the tumor microenvironment, and these activated CD8-positive T-lymphocytes attack not only the tumors but also cause immune-related adverse events (irAEs) (29).

Several studies have consistently reported that concomitant baseline medications using corticosteroids, PPIs, and antibiotics have a negative impact on ICI treatment. For instance, Cortellini et al. (18) reported that in patients with advanced cancer treated with single-agent ICIs (*n* = 1012) as a first- and later-line treatment, baseline corticosteroids, PPIs, and antibiotics were associated with worse clinical outcomes. In another study, Cortellini et al. (19) also showed that in patients with advanced NSCLC who received pembrolizumab (*n* = 950) as a first-line treatment, baseline corticosteroids, PPIs, and antibiotics were associated with worse clinical outcomes. In the present study, six patients received metformin, two patients received fibrates, and eighteen patients received statins, and these medications were not analyzed because of the small number of patients. In contrast, 97 patients received NSAIDs. A sub-analysis to evaluate the association between co-administration of NSAIDs and survival outcomes revealed a significant association between NSAIDs and OS (log-rank test, *P* = 0.031). However, there was multicollinearity between PPIs and NSAIDs because PPIs were prescribed for prophylaxis of NSAID-induced gastric ulcers. Additionally, there have been no reports on the relationship between baseline concomitant NSAIDs and survival outcomes of ICIs (18, 30, 31). Therefore, NSAIDs were not used as a drug-based prognostic score in this study.

To account for the presence of a delay in benefit that may be typical with immunotherapy, we excluded some patients who discontinued the treatment 6 weeks after ICI initiation. In addition, a pivotal phase III study showed a crossing of the PFS curves between nivolumab and docetaxel in patients with advanced non-squamous NSCLC (32). Overall, the present study may be driven by certain subgroups of patients who have a delay in the benefit of ICI treatment. Other reasons for choosing a 6-week period included the occurrence rate of irAEs and the timing of computed tomography. The occurrence rate of irAEs was approximately 50% within 6 weeks of ICI treatment (26, 33). Imaging evaluations were conducted every 6–8 weeks, starting at treatment initiation.

This study has some limitations. First, this was a retrospective observational study; therefore, the presence of an information bias cannot be excluded. In addition, medical records used in this study were heterogeneous between physicians and may not have been recorded completely, especially those of the baseline medications—we concomitantly used medical records written by pharmacists. Methodologically, the three groups did not match, and the sample size was unequal; therefore, to reduce the effect of potential confounding factors associated with observational studies and clinical differences in patient characteristics, we used multivariable analyses. Nevertheless, unmeasured confounders such as other baseline medications, PD-L1 expression, and post-treatment of ICI were not controlled during the multivariable analyses, which is a major limitation of our study since controlling these could have affected the results. Second, our data were derived from a single-center, and the sample size was relatively small compared with those of previous studies that created a drug-based prognostic score for the first time (16, 17). Third, the present study did not evaluate the onset of irAEs. According to a previous study, irAEs were associated with poor survival outcomes (33). In contrast, Miura et al. (31) reported that in Japanese patients with advanced NSCLC treated with nivolumab or pembrolizumab (*n* = 300), baseline medications were not significantly associated with the onset of irAEs. Fourth, the present study did not evaluate the smoking status because of its retrospective nature. Finally, the present study mainly evaluated second- or later-line treatments. The current standard first-line treatment for advanced NSCLC is a combination of ICI and platinum doublet; therefore, the clinical significance of this study is relatively weak. Overall, the findings of this study should be validated in a large-scale multicenter study with an adequate sample size of patients treated with a variety of baseline medications that affect the gut microbiota.

In conclusion, the findings suggest that baseline medications plus NLR could lead to progressively shorter survival outcomes in patients with advanced NSCLC treated with nivolumab or pembrolizumab in clinical practice. Our data provide preliminary evidence for prognostic score-driven baseline medication and routinely available blood cell counts at baseline in Japanese patients with advanced NSCLC and suggest that the prognostic scores could be used as a prognostic index for predicting survival outcomes in clinical practice. These findings are likely to be translated to other Asian populations, highlighting the need for additional research in this field. Finally, since the present study only employed a training cohort, our prognostic score-driven baseline medications and NLR should be confirmed using a large sample size as a validation cohort.

## Data Availability Statement

The raw data supporting the conclusions of this article will be made available by the authors, without undue reservation.

## Ethics Statement

The studies involving human participants were reviewed and approved by National Cancer Center Institutional Review Board (approval number: 2019-305). The ethics committee waived the requirement of written informed consent for participation.

## Author Contributions

HK: Concept and design. TO, HK, and SE: acquisition of data (acquired data and managed patients). TO, HK, and AH: Data analysis and interpretation. TO and HK: Writing, reviewing, and revising the manuscript. TN: Supervision of the study. All authors contributed to manuscript revision and read and approved the submitted version.

## Funding

This work was supported in part by the Foundation for Promotion of Cancer Research in Japan and the Research Foundation for Pharmaceutical Sciences in Japan. The funders had no role in the design of the study; the collection, analysis, and interpretation of the data; the writing of the manuscript; and the decision to submit the manuscript for publication.

## Conflict of Interest

YO received research funding from Kissei, Dainippon-Sumitomo, Ignyta, LOXO, AstraZeneca, Taiho Pharmaceutical, Chugai, Lilly, Ono Pharmaceutical, Bristol-Myers Squibb, Pfizer, MSD, Kyorin, Takeda, and Novartis and received honoraria from AstraZeneca, Taiho Pharmaceutical, Chugai, Lilly, Ono Pharmaceutical, Bristol-Myers Squibb, Pfizer, MSD, Kyorin, Takeda, Novartis, Celltrion, Amgen, and Boehringer Ingelheim. TN received research funding from Astellas Pharma, Chugai, Daiichi Sankyo, Otsuka Pharmaceutical, Sanofi, and Shionogi.

The remaining authors declare that the research was conducted in the absence of any commercial or financial relationships that could be construed as a potential conflict of interest.

## Publisher’s Note

All claims expressed in this article are solely those of the authors and do not necessarily represent those of their affiliated organizations, or those of the publisher, the editors and the reviewers. Any product that may be evaluated in this article, or claim that may be made by its manufacturer, is not guaranteed or endorsed by the publisher.

## Abbreviations

CI, confidence interval; ECOG PS, Eastern Cooperative Oncology Group performance status; HR, hazard ratio; ICI, immune checkpoint inhibitor; irAE, immune-related adverse event; IQR, interquartile range; NLR, neutrophil-to-lymphocyte ratio; NSAIDs, nonsteroidal anti-inflammatory drugs; NSCLC, non-small-cell lung cancer; OS, overall survival; PD-1, programmed cell death protein 1; PFS, progression-free survival; PPI, proton pump inhibitors.

## References

[1] Global Burden of Disease Cancer Collaboration. Global, Regional, and National Cancer Incidence, Mortality, Years of Life Lost, Years Lived With Disability, and Disability-Adjusted Life-Years for 29 Cancer Groups, 1990 to 2016: A Systematic Analysis for the Global Burden of Disease Study. JAMA Oncol (2018) 4(11):1553–68. doi: 10.1001/jamaoncol.2018.2706 PMC6248091 29860482

[2] Masnoon N Shakib S Kalisch-Ellett L Caughey GE. What is Polypharmacy? A Systematic Review of Definitions. BMC Geriatr (2017) 17(1):230. doi: 10.1186/s12877-017-0621-2 29017448 PMC5635569

[3] Hakozaki T Hosomi Y Shimizu A Kitadai R Mirokuji K Okuma Y. Polypharmacy as a Prognostic Factor in Older Patients With Advanced non-Small-Cell Lung Cancer Treated With Anti-PD-1/PD-L1 Antibody-Based Immunotherapy. J Cancer Res Clin Oncol (2020) 146(10):2659–68. doi: 10.1007/s00432-020-03252-4 PMC11804739 32462298

[4] Kubo T Ninomiya T Hotta K Kozuki T Toyooka S Okada H. Study Protocol: Phase-Ib Trial of Nivolumab Combined With Metformin for Refractory/Recurrent Solid Tumors. Clin Lung Cancer (2018) 19(6):e861–4. doi: 10.1016/j.cllc.2018.07.010 30172698

[5] Chamoto K Chowdhury PS Kumar A Sonomura K Matsuda F Fagarasan S. Mitochondrial Activation Chemicals Synergize With Surface Receptor PD-1 Blockade for T Cell-Dependent Antitumor Activity. Proc Natl Acad Sci USA (2017) 114(5):E761–70. doi: 10.1073/pnas.1620433114 PMC5293087 28096382

[6] Chowdhury PS Chamoto K Kumar A Honjo T. PPAR-Induced Fatty Acid Oxidation in T Cells Increases the Number of Tumor-Reactive CD8+ T Cells and Facilitates Anti-PD-1 Therapy. Cancer Immunol Res (2018) 6(11):1375–87. doi: 10.1158/2326-6066.CIR-18-0095 30143538

[7] Eikawa S Nishida M Mizukami S Yamazaki C Nakayama E Udono H. Immune-Mediated Antitumor Effect by Type 2 Diabetes Drug, Metformin. Proc Natl Acad Sci USA (2015) 112(6):1809–14. doi: 10.1073/pnas.1417636112 PMC4330733 25624476

[8] Arbour KC Mezquita L Long N Rizvi H Auclin E Ni A. Impact of Baseline Steroids on Efficacy of Programmed Cell Death-1 and Programmed Death-Ligand 1 Blockade in Patients With Non-Small-Cell Lung Cancer. J Clin Oncol (2018) 36(28):2872–8. doi: 10.1200/JCO.2018.79.0006 30125216

[9] Ricciuti B Dahlberg SE Adeni A Sholl LM Nishino M Awad MM. Immune Checkpoint Inhibitor Outcomes for Patients With Non-Small-Cell Lung Cancer Receiving Baseline Corticosteroids for Palliative Versus Nonpalliative Indications. J Clin Oncol (2019) 37(22):1927–34. doi: 10.1200/JCO.19.00189 31206316

[10] Libert C Dejager L. How Steroids Steer T Cells. Cell Rep (2014) 7(4):938–9. doi: 10.1016/j.celrep.2014.04.041 24856295

[11] Derosa L Hellmann MD Spaziano M Halpenny D Fidelle M Rizvi H. Negative Association of Antibiotics on Clinical Activity of Immune Checkpoint Inhibitors in Patients With Advanced Renal Cell and non-Small-Cell Lung Cancer. Ann Oncol (2018) 29(6):1437–44. doi: 10.1093/annonc/mdy103 PMC6354674 29617710

[12] Routy B Le Chatelier E Derosa L Duong CPM Alou MT Daillère R. Gut Microbiome Influences Efficacy of PD-1-Based Immunotherapy Against Epithelial Tumors. Science (2018) 359(6371):91–7. doi: 10.1126/science.aan3706 29097494

[13] Imhann F Vich Vila A Bonder MJ Lopez Manosalva AG Koonen DPY Fu J. The Influence of Proton Pump Inhibitors and Other Commonly Used Medication on the Gut Microbiota. Gut Microbes (2017) 8(4):351–8. doi: 10.1080/19490976.2017.1284732 PMC5570416 28118083

[14] Chalabi M Cardona A Nagarkar DR Dhawahir Scala A Gandara DR Rittmeyer A. Efficacy of Chemotherapy and Atezolizumab in Patients With Non-Small-Cell Lung Cancer Receiving Antibiotics and Proton Pump Inhibitors: Pooled *Post Hoc* Analyses of the OAK and Poplar Trials. Ann Oncol (2020) 31(4):525–31. doi: 10.1016/j.annonc.2020.01.006 32115349

[15] Omori M Okuma Y Hakozaki T Hosomi Y. Statins Improve Survival in Patients Previously Treated With Nivolumab for Advanced Non-Small-Cell Lung Cancer: An Observational Study. Mol Clin Oncol (2019) 10(1):137–43. doi: 10.3892/mco.2018.1765 PMC6313973 30655989

[16] Buti S Bersanelli M Perrone F Tiseo M Tucci M Adamo V. Effect of Concomitant Medications With Immune-Modulatory Properties on the Outcomes of Patients With Advanced Cancer Treated With Immune Checkpoint Inhibitors: Development and Validation of a Novel Prognostic Index. Eur J Cancer (2021) 142:18–28. doi: 10.1016/j.ejca.2020.09.033 33212418

[17] Buti S Bersanelli M Perrone F Bracarda S Di Maio M Giusti R. Predictive Ability of a Drug-Based Score in Patients With Advanced Non-Small-Cell Lung Cancer Receiving First-Line Immunotherapy. Eur J Cancer (2021) 150:224–31. doi: 10.1016/j.ejca.2021.03.041 33934059

[18] Cortellini A Tucci M Adamo V Stucci LS Russo A Tanda ET. Integrated Analysis of Concomitant Medications and Oncological Outcomes From PD-1/PD-L1 Checkpoint Inhibitors in Clinical Practice. J Immunother Cancer (2020) 8(2):e001361. doi: 10.1136/jitc-2020-001361 33154150 PMC7646355

[19] Cortellini A Di Maio M Nigro O Leonetti A Cortinovis DL Aerts JG. Differential Influence of Antibiotic Therapy and Other Medications on Oncological Outcomes of Patients With non-Small Cell Lung Cancer Treated With First-Line Pembrolizumab Versus Cytotoxic Chemotherapy. J Immunother Cancer (2021) 9(4):e002421. doi: 10.1136/jitc-2021-002421 33827906 PMC8031700

[20] Rebuzzi SE Signori A Banna GL Maruzzo M De Giorgi U Pedrazzoli P. Inflammatory Indices and Clinical Factors in Metastatic Renal Cell Carcinoma Patients Treated With Nivolumab: The Development of a Novel Prognostic Score (Meet-URO 15 Study). Ther Adv Med Oncol (2021) 13:17588359211019642. doi: 10.1177/17588359211019642 34046089 PMC8135208

[21] Sacdalan DB Lucero JA Sacdalan DL. Prognostic Utility of Baseline Neutrophil-to-Lymphocyte Ratio in Patients Receiving Immune Checkpoint Inhibitors: A Review and Meta-Analysis. Onco Targets Ther (2018) 11:955–65. doi: 10.2147/OTT.S153290 PMC5827677 29503570

[22] Zhang N Jiang J Tang S Sun G. Predictive Value of Neutrophil-Lymphocyte Ratio and Platelet-Lymphocyte Ratio in Non-Small Cell Lung Cancer Patients Treated With Immune Checkpoint Inhibitors: A Meta-Analysis. Int Immunopharmacol (2020) 85:106677. doi: 10.1016/j.intimp.2020.106677 32531712

[23] University of Bern, Institute of Social and Preventive Medicine. STROBE Statement (2009). Available at: https://www.strobe-statement.org/index.php?id=strobe-home (Accessed August 2, 2021).

[24] Kawazoe H Mori N Ido S Uozumi R Tsuneoka K Takeuchi A. Liquid Formulation of Gemcitabine Increases Venous Pain in Patients With Cancer: A Retrospective Study. Clin Ther (2020) 42(4):712–9. doi: 10.1016/j.clinthera.2020.02.010 32160969

[25] Egami S Kawazoe H Hashimoto H Uozumi R Arami T Sakiyama N. Peripheral Blood Biomarkers Predict Immune-Related Adverse Events in Non-Small Cell Lung Cancer Patients Treated With Pembrolizumab: A Multicenter Retrospective Study. J Cancer (2021) 12(7):2105–12. doi: 10.7150/jca.53242 PMC7974524 33754009

[26] Egami S Kawazoe H Hashimoto H Uozumi R Arami T Sakiyama N. Absolute Lymphocyte Count Predicts Immune-Related Adverse Events in Patients With Non-Small-Cell Lung Cancer Treated With Nivolumab Monotherapy: A Multicenter Retrospective Study. Front Oncol (2021) 11:618570. doi: 10.3389/fonc.2021.618570 34123782 PMC8190379

[27] Eisenhauer EA Therasse P Bogaerts J Schwartz LH Sargent D Ford R. New Response Evaluation Criteria in Solid Tumours: Revised RECIST Guideline (Version 1. 1) Eur J Cancer (2009) 45(2):228–47. doi: 10.1016/j.ejca.2008.10.026 19097774

[28] Farrell B Pottie K Thompson W Boghossian T Pizzola L Rashid FJ. Deprescribing Proton Pump Inhibitors: Evidence-Based Clinical Practice Guideline. Can Fam Physician (2017) 63(5):354–64. PMC5429051 28500192

[29] Chen D Chen DS Irving BA Hodi FS. Molecular Pathways: Next-Generation Immunotherapy–Inhibiting Programmed Death-Ligand 1 and Programmed Death-1. Clin Cancer Res (2012) 18(24):6580–7. doi: 10.1158/1078-0432.CCR-12-1362 23087408

[30] Wang DY McQuade JL Rai RR Park JJ Zhao S Ye F. The Impact of Nonsteroidal Anti-Inflammatory Drugs, Beta Blockers, and Metformin on the Efficacy of Anti-PD-1 Therapy in Advanced Melanoma. Oncologist (2020) 25(3):e602–5. doi: 10.1634/theoncologist.2019-0518 PMC7066699 32162820

[31] Miura K Sano Y Niho S Kawasumi K Mochizuki N Yoh K. Impact of Concomitant Medication on Clinical Outcomes in Patients With Advanced non-Small-Cell Lung Cancer Treated With Immune Checkpoint Inhibitors: A Retrospective Study. Thorac Cancer (2021) 12(13):1983–94. doi: 10.1111/1759-7714.14001 PMC8258365 33990133

[32] Borghaei H Paz-Ares L Horn L Spigel DR Steins M Ready NE. Nivolumab Versus Docetaxel in Advanced Nonsquamous Non-Small-Cell Lung Cancer. N Engl J Med (2015) 373(17):1627–39. doi: 10.1056/NEJMoa1507643 PMC5705936 26412456

[33] Haratani K Hayashi H Chiba Y Kudo K Yonesaka K Kato R. Association of Immune-Related Adverse Events With Nivolumab Efficacy in Non-Small-Cell Lung Cancer. JAMA Oncol (2018) 4(3):374–8. doi: 10.1001/jamaoncol.2017.2925 PMC6583041 28975219

