# Supplementary material for: Prognostic Value of Baseline Medications Plus Neutrophil-to-Lymphocyte Ratio in the Effectiveness of Nivolumab and Pembrolizumab in Patients With Advanced Non-Small-Cell Lung Cancer: A Retrospective Study
Source: Front Oncol. 2021 Nov 8;11:770268. doi: 10.3389/fonc.2021.770268 (PMC8606521; doi:10.3389/fonc.2021.770268)
Supplement: Supplementary file 1 [file Table_1.docx]

Supplementary Material

# Supplementary Tables

**Table S1.** Univariable and multivariable Cox proportional hazard ratio of the drug-based prognostic score for overall survival.

|  |  |  |  |  | Univariable analysis | |  | Multivariable analysis | |
| --- | --- | --- | --- | --- | --- | --- | --- | --- | --- |
| Variable | | No. | Event | Censored | HR (95% CI) | *P*-Value |  | HR (95% CI) | *P*-Value |
| Group | Poor-prognosis | 31 | 21 | 10 | 1.55 (0.95–2.42) | 0.064 |  | 1.37 (0.80–2.26) | 0.239 |
|  | Intermediate-prognosis | 110 | 61 | 49 | 1.13 (0.81–1.58) | 0.461 |  | 1.18 (0.82–1.71) | 0.376 |
|  | Good-prognosis | 118 | 60 | 58 | 1 |  |  | 1 |  |
| Age (10-year intervals) | | – | – | – | 0.91 (0.79–1.06) | 0.227 |  | 0.97 (0.83–1.13) | 0.657 |
| ECOG PS | 2 | 28 | 21 | 7 | 2.75 (1.68–4.29) | <0.001 |  | 2.37 (1.41–3.78) | <0.001 |
|  | 0–1 | 231 | 121 | 110 | 1 |  |  | 1 |  |
| Treatment line | Later-line | 195 | 116 | 79 | 1.66 (1.10–2.60) | 0.020 |  | 1.56 (1.03–2.45) | 0.046 |
|  | First-line | 64 | 26 | 38 | 1 |  |  | 1 |  |

Abbreviations: ECOG PS, Eastern Cooperative Oncology Group performance status; HR, hazard ratio; CI, confidence interval.

**Table S2.** Univariable and multivariable Cox proportional hazard ratio of the neutrophil-to-lymphocyte ratio for overall survival.

|  |  |  |  |  | Univariable analysis | |  | Multivariable analysis | |
| --- | --- | --- | --- | --- | --- | --- | --- | --- | --- |
| Variable | | No. | Event | Censored | HR (95% CI) | *P*-Value |  | HR (95% CI) | *P*-Value |
| NLR | ≥3 | 166 | 96 | 70 | 1.42 (1.01–2.04) | 0.049 |  | 1.38 (0.97–1.99) | 0.076 |
|  | <3 | 93 | 46 | 47 | 1 |  |  | 1 |  |
| Age (10-year intervals) | | – | – | – | 0.91 (0.79–1.06) | 0.227 |  | 0.96 (0.83–1.13) | 0.646 |
| ECOG PS | 2 | 28 | 21 | 7 | 2.75 (1.68–4.29) | <0.001 |  | 2.43 (1.46–3.86) | <0.001 |
|  | 0–1 | 231 | 121 | 110 | 1 |  |  | 1 |  |
| Treatment line | Later-line | 195 | 116 | 79 | 1.66 (1.10–2.60) | 0.020 |  | 1.62 (1.07–2.54) | 0.028 |
|  | First-line | 64 | 26 | 38 | 1 |  |  | 1 |  |

Abbreviations: NLR, neutrophil-to-lymphocyte ratio; ECOG PS, Eastern Cooperative Oncology Group performance status; HR, hazard ratio; CI, confidence interval.
